# Innovative use of a long transparent cap for closure of large duodenal defects post endoscopic full-thickness resection: a case report

**DOI:** 10.1016/j.igie.2025.07.007

**Published:** 2025-07-25

**Authors:** Nan Dai, Chang-Qing Guo, Shan-Shan Zhu, Saif Ullah, Xin-Guang Cao

**Affiliations:** Department of Gastroenterology, The First Affiliated Hospital of Zhengzhou University, Zhengzhou, Henan, China

## Abstract

Securing large duodenal defects after endoscopic full-thickness resection (EFTR) is challenging because of anatomic constraints. This case report describes an innovative closure technique using a modified long transparent cap in a 48-year-old man who underwent EFTR for a 2.0 × 2.5-cm duodenal bulb gastrointestinal stromal tumor, resulting in a 1.5 × 2.5-cm full-thickness defect. Titanium clips closure failed. A long ligation device cap (inner diameter 0.9 cm, outer diameter 1.1 cm) was used to suction opposing mucosal edges, enabling precise titanium clip placement under endoscopic guidance. The cap-assisted technique achieved secure defect closure. Margin-negative resection was confirmed, and the patient recovered without adverse events. To our knowledge, we report the first use of a long transparent cap that helped successfully close a large duodenal defect following EFTR, offering a practical alternative in anatomically challenging locations or resource-limited settings.

## Introduction

Gastrointestinal stromal tumors (GISTs) represent the most common mesenchymal neoplasms of the digestive tract, with duodenal GISTs constituting 4% to 5% of cases.^1^ Although endoscopic advances have enabled a gradual transition from surgical to endoscopic management of duodenal GISTs, achieving secure closure after endoscopic full-thickness resection (EFTR) remains a critical challenge especially if the full-thickness resection device or endoscopic suturing systems are not available. This is underscored by substantial closure-related adverse event risks, with perforation and bleeding rates exceeding 50% for lesions ≥30 mm.^2^ Consequently, postendoscopic duodenal perforation is recognized as one of the most-severe adverse events^3^; this vulnerability stems from the thin muscularis propria and retroperitoneal anatomy.

There are limitations to existing closure methods: through-the-scope clips (TTSCs) fail to close defects greater than 1 cm^4^; newer TTSCs like the MANTIS clip (Boston Scientific, Marlborough, Mass, USA) and Dual Action Tissue clip (Micro-Tech Endoscopy, Nanjing, China) can close defects up to 3 or 5 cm, respectively, but are more expensive; nylon loop systems are impeded by narrow luminal anatomy,^5^^,^^6^ the Over-The-Scope Clip (OTSC; Ovesco Endoscopy AG, Tübingen, Germany) is costly and closes defects up to 3 cm in size, and suturing systems (OverStitch; Boston Scientific) are also costly and not widely available, particularly in resource-constrained settings.^7^

To address these limitations, we used a long transparent ligation device cap (notably longer than standard caps) to enhance suction efficacy. Using a dedicated suction technique, we drew mucosa bilaterally into the cap, enabling the clips to grasp greater tissue volume for secure closure—a cost-effective and technically straightforward approach.

## Case description

**Figure 1:**
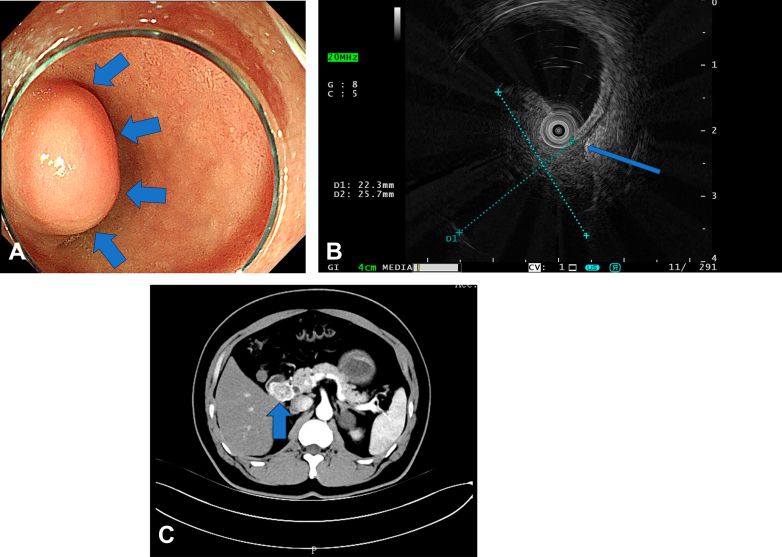
**A,** Endoscopy shows a mass in the duodenal bulb (*arrows*). **B,** Endoscopic ultrasound image showing 22.3 × 25.7-mm hypoechoic lesion arising from muscularis propria layer (*arrow*). **C,** Computed tomography image suggests a stromal tumor (*arrow*) in the duodenal bulb.

A 48-year-old man presented with epigastric pain, no significant family or psychosocial history, no prior abdominal surgeries, or chronic illnesses. Abdominal examination preprocedure revealed a soft, nontender, nondistended abdomen with normal bowel sounds. Ten days after symptom onset, initial esophagogastroduodenoscopy at a local hospital identified a duodenal subepithelial lesion. The patient presented to our institution 1 week later. Endoscopy revealed a 2.0 × 2.5-cm hemispherical protrusion in the duodenal bulb (Fig. 1A), confirmed by endoscopic ultrasonography as a muscularis propria–derived hypoechoic lesion (Fig. 1B). On the same day, contrast-enhanced computed tomography (CT) demonstrated a 32 × 24-mm intraluminal-extraluminal mass consistent with stromal tumor characteristics (Fig. 1C). Following multidisciplinary discussion and patient consent, EFTR was chosen, with preoperative biopsy omitted to avoid potential tissue edema and adhesion, which is based on established clinical practices for resectable GISTs.^5^

**Figure 2:**
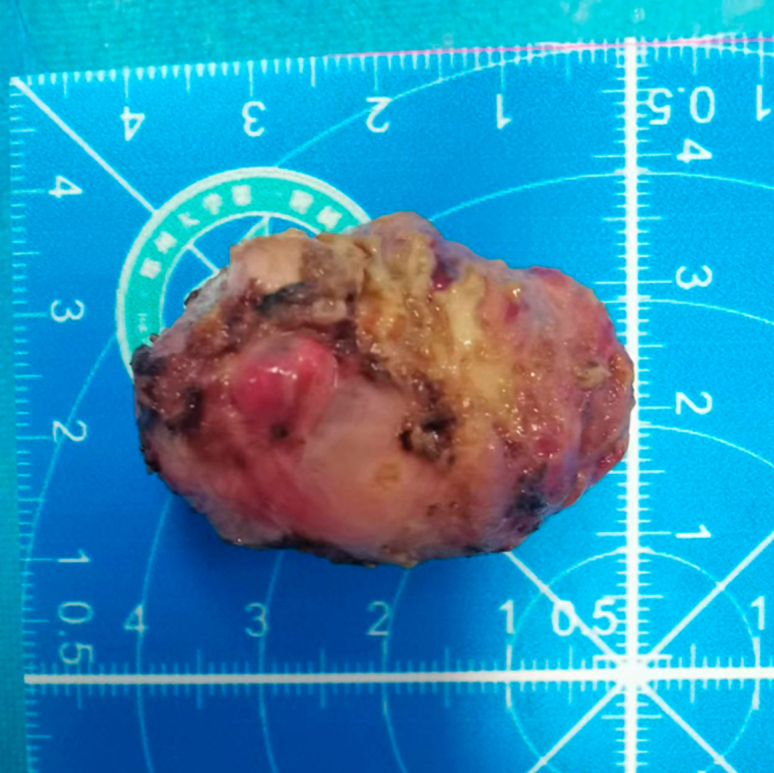
The resected lesion (4 × 2.5 cm).

**Figure 3:**
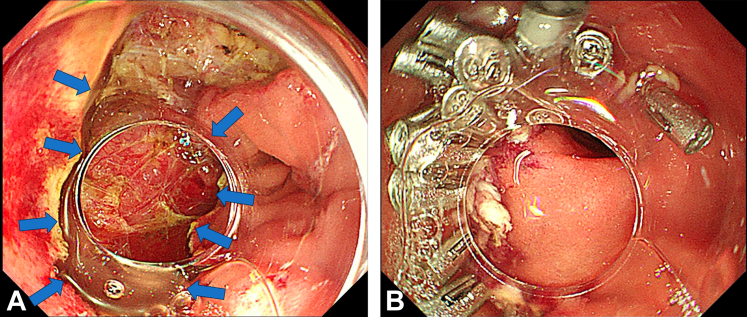
**A,** The full-thickness defect of approximately 1.5 × 2.5 cm after endoscopic resection (*arrows*). **B,** Endoscopic image showing complete wound closure with multiple clips.

On hospital day 3, the procedure was performed with the patient under general anesthesia with endotracheal intubation and the patient in the left lateral decubitus position. During EFTR, a dual knife (KD-650L; Olympus Medical Systems, Tokyo, Japan) was used to incise the mucosal cap covering the lesion, progressively detaching it from the muscularis propria. The resected tissue was sent for pathologic analysis (Fig. 2). Endoscopic view revealed a significant full-thickness defect of 1.5 × 2.5 cm (Fig. 3A). Closure with titanium clips was unfeasible because of the wound size and anatomic constraints; additionally, the narrow space and thin wall also complicated the use of nylon ropes and titanium clips.

**Figure 4:**
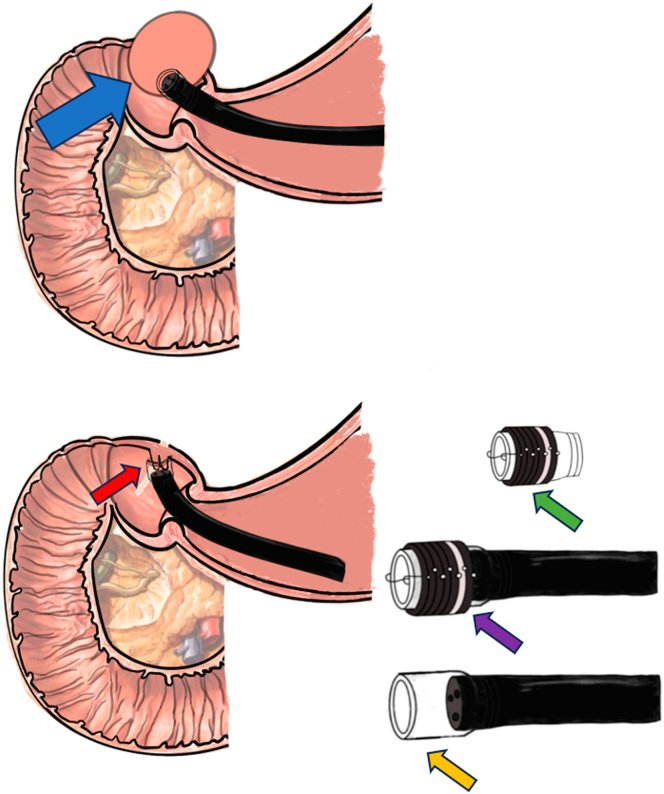
Schematic diagram. Duodenal gastrointestinal stromal tumor (*blue arrow*); long transparent cap and ligation ring from the ligation device (*green arrow*); schematic diagram of the full assembly mounted on the endoscope tip (*purple arrow*); long transparent cap positioned on the endoscope tip with the ligation ring detached (*yellow arrow*); defect closure using clips under suction assistance facilitated by the long transparent cap (*red arrow*).

To address this challenge, we innovatively adapted a long transparent cap (inner diameter 0.9 cm, outer diameter 1.1 cm; Tianjin Tianyi Medical Biomedical Materials Research Co, Ltd, China) from a ligation device to enhance visualization and tissue manipulation. The modified closure protocol involved (1) saline solution irrigation for optimal visualization, (2) controlled suction of opposing mucosal edges into the cap, (3) a total of 24 titanium clips (ROCC-D-26-195; Micro-Tech Co, Ltd) were deployed for defect closure (total procedure time 204 minutes, with wound closure requiring 72 minutes [Fig. 3B, Video 1, available online at www.igiejournal.org]), and (4) closure integrity was confirmed by carbon dioxide (CO_2_) insufflation test demonstrating sustained gastric distension without leakage. Continuous CO_2_ insufflation was maintained at 1.9 ± 0.2 L/min throughout the procedure using an endoscopic insufflation unit (Olympus UCR). Histopathologic examination confirmed margin-negative resection of a duodenal GIST. Immunohistochemistry showed cluster of differentiation 117(+), discovered on GIST-1(+), Ki-67 protein = 1%, with <5 mitoses/50 high-power field, consistent with low-risk stratification National Institutes of Health criteria. Figure 4 provides a schematic diagram of the procedure.

Postoperatively, the patient was placed in a semi-Fowler position with nothing-by-mouth status and nasogastric tube decompression. Continuous monitoring included daily vital signs (temperature, blood pressure, pulse). Intravenous pantoprazole 20 mg daily, prophylactic cefazolin 1 g every 8 hours, and parenteral nutrition were provided. Clinical indicators of adverse events—including hematemesis, fever (>38°C), localized abdominal pain, or peritoneal signs—were vigilantly assessed to detect bleeding, perforation, or infection. No procedure-related adverse events occurred. A postoperative day 4 CT scan showed no perforation, allowing diet advancement to liquids. With subsequent progression to regular diet, the patient was discharged on postoperative day 6 following confirmation of stable recovery. The patient expressed satisfaction with the successful nonsurgical resection of the GIST.

## Discussion

EFTR on duodenal locations poses unique technical challenges, particularly in achieving secure closure of large full-thickness defects. Although established closure systems—including over-the-scope clips and suturing systems—demonstrate efficacy, their limited availability and high cost restrict widespread adoption in Asian countries.^8^ Alternative techniques like TTSCs and purse-string sutures using nylon endoloops with metal clips also face limitations: TTSCs are inadequate for large defects or high-tension sites, whereas purse-string suture requires advanced technical expertise.^9^ Therefore, we present a case using a modified transparent cap from a ligation device to enhance closure efficacy. This technique addresses limitations of existing methods and adds to the literature on minimally invasive management of duodenal GISTs. Previously, this method was applied to gastric GISTs,^10^ and this case marks its first use in duodenal full-thickness defects, to our knowledge. Future studies will compare this technique with OTSCs, OverStitch, and purse-string methods for cost and efficacy, while evaluating learning curves across operator expertise. Notably, limitations of our case report include its being a single case, unknown long-term outcomes (eg, stenosis), and lack of direct comparison to established systems.

In conclusion, this innovative technique serves as a critical adjunct to conventional clipping, enabling endoscopists to independently achieve efficient and safe closure of complex, deep-seated wounds. Especially in the duodenal bulb where larger closure devices are difficult to maneuver and in regions where larger closure devices are not available, alternative methods should be considered. The technique of using a longer cap from a ligation device to suction the mucosal edges into the cap and to clip the defect closed may provide an effective means of closure for larger defects.

## Institutional Review Board approval statement

This study protocol was reviewed and approved by the Ethics Committee of the First Affiliated Hospital of Zhengzhou University (approval No. 2023-KY-1011-002). All procedures complied with the ethical standards of the Declaration of Helsinki and its amendments.

## Patient consent

The patient in this article has given written informed consent to publication of his case details.

## Disclosure

All authors disclosed no financial relationships.

## Supplementary data

Video 1 Endoscopic resection of duodenal submucosal tumor and wound closure with long transparent cap-assisted clip closure technique.
